# The Human Gonadotropin Releasing Hormone Type I Receptor Is a Functional Intracellular GPCR Expressed on the Nuclear Membrane

**DOI:** 10.1371/journal.pone.0011489

**Published:** 2010-07-08

**Authors:** Michelle Re, Macarena Pampillo, Martin Savard, Céléna Dubuc, Craig A. McArdle, Robert P. Millar, P. Michael Conn, Fernand Gobeil, Moshmi Bhattacharya, Andy V. Babwah

**Affiliations:** 1 The Children's Health Research Institute, London, Canada; 2 Lawson Health Research Institute, London, Canada; 3 Department of Obstetrics and Gynaecology, The University of Western Ontario, London, Canada; 4 Department of Physiology and Pharmacology, The University of Western Ontario, London, Canada; 5 Department of Pharmacology, Université de Sherbrooke, Sherbrooke, Canada; 6 Laboratories for Integrated Neuroscience and Endocrinology, Department of Clinical Sciences at South Bristol, University of Bristol, Bristol, United Kingdom; 7 MRC Human Reproductive Sciences Unit, The Queen's Medical Research Institute, Edinburgh, United Kingdom; 8 Oregon National Primate Research Center, Oregon Health and Science University, Beaverton, Oregon, United States of America; University of Oldenburg, Germany

## Abstract

The mammalian type I gonadotropin releasing hormone receptor (GnRH-R) is a structurally unique G protein-coupled receptor (GPCR) that lacks cytoplasmic tail sequences and displays inefficient plasma membrane expression (PME). Compared to its murine counterparts, the primate type I receptor is inefficiently folded and retained in the endoplasmic reticulum (ER) leading to a further reduction in PME. The decrease in PME and concomitant increase in intracellular localization of the mammalian GnRH-RI led us to characterize the spatial distribution of the human and mouse GnRH receptors in two human cell lines, HEK 293 and HTR-8/SVneo. In both human cell lines we found the receptors were expressed in the cytoplasm and were associated with the ER and nuclear membrane. A molecular analysis of the receptor protein sequence led us to identify a putative monopartite nuclear localization sequence (NLS) in the first intracellular loop of GnRH-RI. Surprisingly, however, neither the deletion of the NLS nor the addition of the *Xenopus* GnRH-R cytoplasmic tail sequences to the human receptor altered its spatial distribution. Finally, we demonstrate that GnRH treatment of nuclei isolated from HEK 293 cells expressing exogenous GnRH-RI triggers a significant increase in the acetylation and phosphorylation of histone H3, thereby revealing that the nuclear-localized receptor is functional. Based on our findings, we conclude that the mammalian GnRH-RI is an intracellular GPCR that is expressed on the nuclear membrane. This major and novel discovery causes us to reassess the signaling potential of this physiologically and clinically important receptor.

## Introduction

The gonadotropin releasing hormone receptor (GnRH-R) is a G protein-coupled receptor (GPCR) belonging to the rhodopsin family. In mammals, the type I receptor (GnRH-RI) is expressed in a variety of cell-types including pituitary gonadotropes, T-cells and placental cytotrophoblasts [1], [2]. Ligand-activated GnRH-RI couples to several different G proteins, including G_q/11_, G_s_ and G_i_ [1], [3]–[5]. In humans, GnRH-RI is located at 4q13.1–q21.1 and consists of three exons and two introns that encode a 328 amino acid protein [6], [7].

The mammalian type I GnRH-R is structurally unique among GPCRs, including other GnRH-Rs expressed in non-mammalian and some primate species, in that it does not possess an intracellular tail [6]. The absence of COOH-tail sequences may have resulted in a receptor that lacks sequences necessary for mediating rapid desensitization and internalization as well as strong plasma membrane expression (PME) [8]. In addition, based on biochemical studies (radioligand binding and inositol phosphate formation assays) performed in African Green Monkey Kidney Fibroblast Cells (COS-1) and Human Embryonic Kidney (HEK 293) cells, it has been demonstrated that the presence of a primate-specific lysine residue at position 191 (K191) in the human GnRH-RI contributes to increased internalization kinetics of the receptor, leading to an overall reduction in PME compared to its rat and mouse counterparts [9], [10]. The presence of this primate-specific K191 has been suggested to cause the disruption of a sulfhydryl bridge and subsequent formation of misfolded receptors resulting in the retention of the protein in the endoplasmic reticulum (ER) [11]. The loss of cytoplasmic tail sequences coupled to the presence of K191 are believed to be the result of a recently evolved strategy that has led to decreased PME and greater intracellular localization of GnRH-RI. This strategy provides the cell a very effective mechanism for rapidly regulating cell surface receptor number and hence GnRH-RI-mediated cell signaling events [11].

It is believed that the ER-retained misfolded receptors are eventually degraded by the cell's quality control system [11]. It has also been suggested that part of this retained intracellular pool of receptor provides a source of GnRH-RI needed for rapid availability to the cell without the need for transcription or translation [11]. In addition to these suggestions, we propose that in cells which express both the receptor and ligand, greater intracellular localization of GnRH-RI is perhaps an indication that the cell is evolving towards greater intracrine signaling. To pursue these ideas further, we undertook a detailed spatial and functional characterization of the intracellular receptor. Here we report the human and mouse FLAG-GnRH receptors are expressed intracellularly in two human cell lines, with strong localization to the nuclear membrane. Finally, we also demonstrate that the nuclear membrane localized human GnRH-RI is functional and that receptor stimulation triggers a robust increase in histone H3 acetylation and phosphorylation. These findings reveal that GnRH-RI is a member of a small but growing class of functional nuclear GPCRs and reveals the existence of an entirely novel facet to GnRH-RI signaling within the cell.

## Results

### Preliminary biochemical and spatial analyses of FLAG-hGnRH-RI

**Figure 1:**
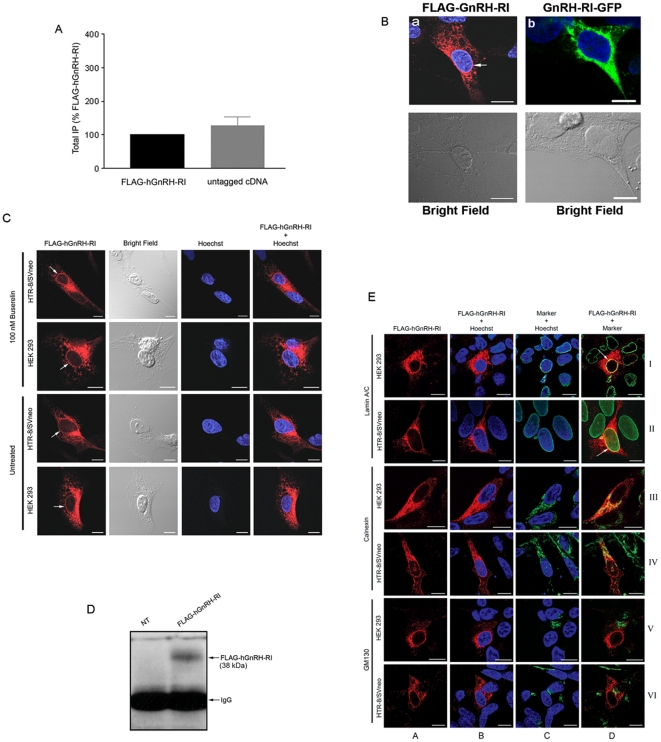
Effect of epitope tag on hGnRH-RI signaling and spatial localization of FLAG-hGnRH-RI. (A) Inositol phosphate (IP) production in response to 100 nM Buserelin was assessed as described in “Materials and Methods”. Data represent three to six independent experiments performed in triplicate and normalized to FLAG-hGnRHRI ± S.E. (B, left) HTR-8/SVneo cells transfected with either FLAG-hGnRH-RI (a) subjected to indirect immunofluorescent staining using affinity purified rabbit anti-FLAG antibody followed by Alexa Fluor 568-conjugated anti-rabbit IgG (*red*) and counterstained with the nuclear dye, Hoechst (*blue*). Note the perinuclear localization of the FLAG-tagged hGnRH-RI (*a*, *arrow*); (B, right) HTR-8/SVneo cell transfected with GnRH-RI-GFP and counterstained with the nuclear dye, Hoechst (*blue*). Note the perinuclear localization of the GnRH-RI-GFP. (C) HTR-8/SVneo and HEK 293 cells transfected with FLAG-hGnRH-RI were subjected to indirect immunofluorescent staining using affinity purified rabbit anti-FLAG antibody followed by Alexa Fluor 568-conjugated anti-rabbit IgG (*red*) and counterstained with Hoechst (*blue*). Note the perinuclear localization of the FLAG-tagged hGnRH-RI seen in both cell lines (*arrows*). Bright field images are shown in the second column from the left. (D) Western blot analysis was performed on the lysates of nuclei isolated from HEK 293 cells overexpressing FLAG-GnRH-RI. The results reveal that the full length hGnRH-RI is expressed on the nuclei of HEK 293 cells. (E) Cells expressing FLAG-hGnRH-RI were subjected to indirect immunofluorescent staining for the receptor (red) as well as the nuclear membrane, endoplasmic reticulum and Golgi (*all shown in green*). Colocalization is seen as yellow staining. Column A: receptor alone; Column B: receptor + Hoescht; Column C: organelle marker (lamin A/C, calnexin or GM130) + Hoescht; Column D: receptor + organelle marker. Column letters and roman numerals used as a coordinate system. hGnRH-RI immunoreactivity co-localized with the nuclear marker, lamin A/C, as seen by the yellow staining (I-D, II-D, *arrows*) as well as with the endoplasmic reticulum marker, calnexin (III-D, IV-D). Less colocalization was seen between the receptor and the Golgi, as shown by less yellow staining (V-D, VI-D). Scale bar = 10 µm.

The HEK 293 cell line is an established cell system for studying regulatory, temporal and spatial characteristics of GPCRs [12]–[14] and based on RT-PCR analysis does not express GnRH-RI (data not shown). HTR-8/SVneo is a trophoblast cell line that is an established system for studying human placentation [2], [15] and previous studies from our lab and elsewhere have demonstrated that trophoblasts, including HTR-8/SVneo cells, express GnRH-RI [1], [2]. In the absence of effective anti-GnRH-RI antibodies we could not examine the spatial distribution of endogenous GnRH-RI in these cells; consequently, we constructed a FLAG amino terminus epitope-tagged receptor (open-reading frame only) for this purpose. Before using this construct to assess the spatial and functional properties of GnRH-RI in these cell lines, the signaling capacity of FLAG-hGnRH-RI was compared to that of an untagged hGnRH-RI cDNA (accession number NM_000406.2). Our data revealed that Buserelin, a metabolically stable GnRH agonist, stimulated similar levels of inositol phosphate (IP) formation by both FLAG-hGnRH-RI- and hGnRH-RI cDNA-expressing HEK 293 cells (Fig. 1A) suggesting that the FLAG epitope does not alter the signaling potential of the receptor. Next, through an immunofluorescence analysis of the FLAG-hGnRH-RI we observed that the receptor was strongly localized intracellularly with a clear perinuclear distribution (Fig. 1B). Immunostaining of non-transfected cells with the FLAG primary and Alexa Fluor 568-conjugated anti-rabbit IgG secondary antibodies yielded unstained cells, suggesting that the perinuclear localization of FLAG-hGnRH-RI is not an artifact of immunostaining (data not shown). Since our spatial analysis thus far was by indirect immunofluorescence (directed against the FLAG epitope), we analyzed the spatial distribution of the hGnRH-RI-GFP chimeric molecule by direct fluorescence and found that this molecule was also strongly distributed in a perinuclear manner (Fig. 1B). This suggested that the perinuclear distribution of FLAG-hGnRH-RI is unlikely the effect of the FLAG epitope. Based on these findings we selected the FLAG-tagged GnRH-RI (human and mouse) for all subsequent studies. The FLAG-GnRH-RI DNA was introduced into HEK 293 cells by calcium-phosphate transfection, while it was introduced into HTR-8/SVneo cells by electroporation. Transfection efficiency of the HEK 293 cells was always greater than 75%, while that for HTR-8/SVneo cells was closer to 25% (data not shown).

### Spatial characterization of GnRH-RI in HEK 293 and HTR-8/SVneo cells

Immunofluorescence studies performed on agonist (Buserelin) treated and untreated Triton X 100-permeabilized cells, using the anti-FLAG antibody clearly revealed that the spatial localization of FLAG-hGnRH-RI is identical in both HEK 293 and HTR-8/SVneo cells (Fig. 1C) and that agonist stimulation did not affect the cellular distribution (Fig. 1C). The presence of the receptor was visually undetectable at the plasma membrane but was strongly localized intracellularly in 100% of the transfected cells. While the receptor was distributed intracellularly throughout the cell there were two intracellular regions in which expression was very high. In 100% of all transfected cells, one region corresponded to a large part of the cytoplasm, generally located to one side of the nucleus only, while in about 75–95% of these cells, the receptor was also strongly observed at the perinuclear region (Fig. 1C). To test the possibility that the perinuclear-localized receptor was a mislocalized truncated molecule, we western-blotted lysates of nuclei isolated from HEK 293 cells overexpressing FLAG-GnRH-RI and observed that it is in fact the full-length receptor that is expressed and localized to this region (Fig. 1D).

To characterize the spatial localization of the receptor in greater detail we examined the location of the receptor relative to calnexin, GM130 and lamin A/C; these are organellar markers for the ER, Golgi and the nuclear membrane, respectively. Our results revealed that a large fraction of the cytoplasmic pool of receptors localized to the ER (Fig. 1E) while the perinuclear pool localized to the nuclear membrane (Fig. 1E). Receptor localization to the Golgi was also visually detectable, however, not as readily as it was in the ER (Fig. 1E). In all cases no differences were observed between the HEK 293 and HTR-8/SVneo cells (Fig. 1E). Since we consistently showed no differences in the localization of the receptor in the two cell lines most of the spatial studies were conducted in the HTR-8/SVneo cell line only.

### Spatial characterization of GnRH-RI, compared to mGluR5a and β2AR, in HTR-8/SVneo cells

**Figure 2:**
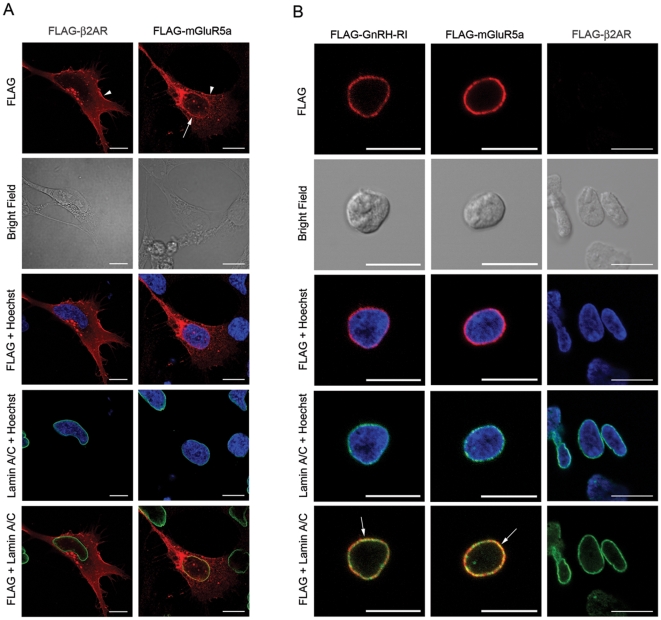
Spatial localization of FLAG-mGluR5a, FLAG-β_2_AR and FLAG-hGnRH-RI. (A) HTR-8/SVneo cells transfected with either FLAG-mGluR5a or FLAG-β_2_AR were subjected to indirect immunofluorescent staining for the receptor (*red*) as well as the nuclear membrane marker lamin A/C (*green*). Cells were counterstained with Hoechst (*blue*). Note the perinuclear localization of the FLAG-tagged mGluR5a *(arrow*) and the plasma membrane localization of both FLAG-mGluR5a and FLAG-β_2_AR (*arrowheads*). Bright field images are shown in the second row from top. (B) Isolated nuclei from HEK293 cells expressing FLAG-hGnRH-RI, FLAG-mGluR5a or FLAG-β_2_AR were subjected to indirect immunofluorescent staining for the receptor (*red*) as well as the nuclear membrane marker lamin A/C (*green*). Cells were counterstained with Hoechst (*blue*). Note the colocalization of both FLAG-hGnRH-RI and FLAG-mGluR5a with the inner nuclear membrane marker lamin A/C (*yellow, arrows*). FLAG-β_2_AR was not localized to the nuclear membrane. Bright field images are shown in the second row from top. Scale bar = 10 µm.

Next, we compared the distribution pattern of FLAG-hGnRH-RI in HTR-8/SVneo cells to that of FLAG-mGluR5a (metabotropic glutamate receptor 5a) and FLAG-β2AR (β2-adrenergic receptor) in HTR-8/SVneo cells (Fig. 2A). Both receptors are GPCRs and mGluR5a has been well characterized as a nuclear GPCR that is expressed on the nuclear membrane [16], [17] while the β2AR has only been detected on the plasma membrane and in the cytoplasm [18], [19]. Immunofluorescence studies on agonist (quisqualate for mGluR5a and isoproterenol for β2AR) treated cells permeabilized prior to their incubation in anti-FLAG antibody revealed that mGluR5a colocalized with lamin A/C at the nuclear membrane in a pattern identical to that of hGnRH-RI and lamin A/C, while as expected, β2AR was strongly expressed at the plasma membrane (Fig. 2A). β2AR and mGluR5a were also seen intracellularly on vesicles as well as evenly distributed throughout the rest of the cytoplasm. However, unlike mGluR5a, β2AR was completely absent at the nuclear membrane. Interestingly, unlike FLAG-mGluR5a and FLAG-β2AR, the cytoplasmic distribution of FLAG-hGnRH-RI was often more strongly localized to one side of the nucleus, rather than throughout the cell (Fig. 2A). To test whether the differences in the spatial distribution were in part due to relative expression levels between the receptors, we performed SYBR green real-time PCR analysis of receptor gene expression in HEK 293 cells and found that all the receptors were expressed at similar levels (data not shown), suggesting that differential expression is not the underlying cause for these observations.

### Spatial characterization of GnRH-RI on isolated nuclei

In addition to looking at the localization of GnRH-RI in whole cells, we also performed immunolocalization studies on intact nuclei isolated from HEK 293 cells that were previously transfected with FLAG-hGnRH-RI or FLAG-mGluR5a. The purpose of these studies was to determine whether the apparent nuclear localization of hGnRH-RI was real or was a visual aberration due to the result of hGnRH-RI being overexpressed at the perinuclear region. If this were the case, it was possible that lamin A/C might have only appeared to colocalize with the pool of receptor molecules in the adjoining perinuclear region. Thus by stripping away the cytoplasmic pool of receptor, we could more confidently assess receptor localization on the nuclear membrane. The results from these experiments revealed that in the absence of the cytosolic components, as confirmed by visual inspection and a lack of Golgi and ER staining, both FLAG-hGnRH-RI and FLAG-mGluR5a were still strongly detectable at the nuclear membrane and colocalized with lamin A/C (Fig. 2B). Furthermore, an analysis of nuclei isolated from cells expressing FLAG-β2AR did not show any receptor on the nuclear membrane (Fig. 2B). These results strongly demonstrate that the nuclear membrane localization of hGnRH-RI is real and not the result of a visual aberration due to overexpression of the receptor in the perinuclear region.

### Characterization of NLS located in GnRH-RI protein sequence

**Figure 3:**
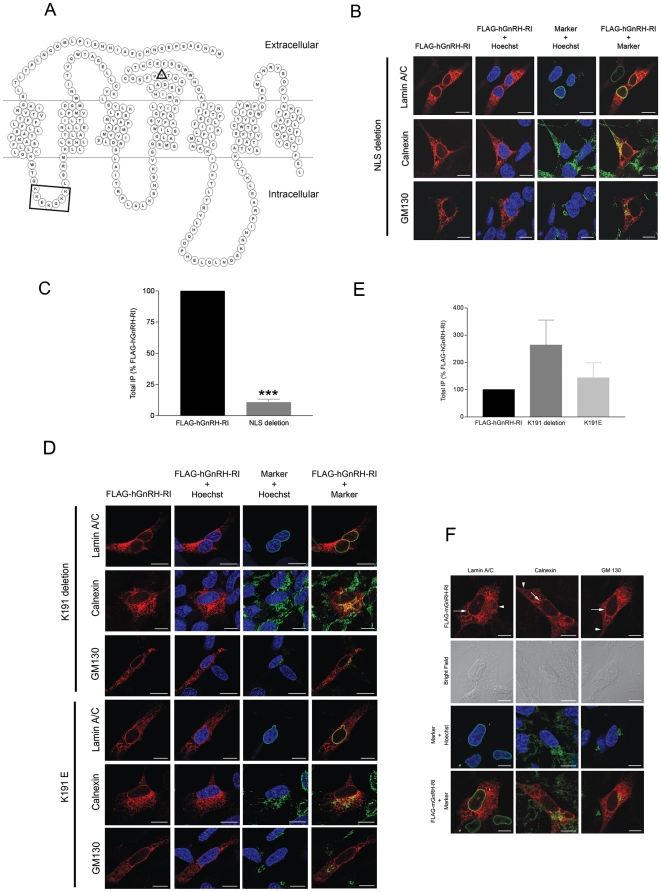
Effect of putative NLS and lysine 191 hGnRH-RI mutants on receptor localization and signaling and spatial localization of FLAG-mGnRH-RI in HTR-8/SVneo cells. (A) Schematic of the human GnRH-RI showing the positions of the lysine 191 residue (triangle) and the location of the putative NLS (square). (B) HTR-8/SVneo cells expressing FLAG-hGnRH-RI in which the putative NLS was deleted were subjected to indirect immunofluorescent staining for the receptor (*red*) as well as the markers for the nuclear membrane, endoplasmic reticulum and Golgi (*shown in green*). Cells were counterstained with Hoechst (*blue*). The NLS deletion mutant showed the same phenotype as the wild-type receptor, showing strong colocalization with lamin A/C and calnexin and less colocalization with GM130. (C) IP formation data represent seven independent experiments performed in triplicate and normalized to FLAG-hGnRHRI ± S.E. ***, p<0.001 versus IP formation of FLAG-hGnRH-RI. (D) HTR-8/SVneo cells expressing FLAG-hGnRH-RI in which lysine 191 was deleted or mutated to a glutamic acid residue (K191E) were subjected to indirect immunofluorescent staining for the receptor (*red*) as well as the markers for the nuclear membrane, endoplasmic reticulum and Golgi (*shown in green*). Cells were counterstained with Hoechst (*blue*). K191 deletion and K191E mutants showed the same phenotype as the wild-type receptor, showing strong colocalization with lamin A/C and calnexin and less colocalization with GM130. (E) IP formation data represent 6–7 independent experiments performed in triplicate and normalized to FLAG-hGnRHRI ± S.E. (F) HTR-8/SVneo cells expressing mGnRH-RI were subjected to indirect immunofluorescent staining for the receptor (*red*) as well as the markers for the nuclear membrane, endoplasmic reticulum and Golgi (*shown in green*). Cells were counterstained with Hoechst (*blue*). FLAG-mGnRH-RI showed similar perinuclear localization (arrows) to FLAG-hGnRH-RI with strong colocalization with lamin A/C and less colocalization with GM130. However, less colocalization was seen between the FLAG-mGnRH-RI and calnexin than was seen with the FLAG-hGnRH-RI. As well, there was evidence of plasma membrane localization (arrowheads) with FLAG-mGnRH-RI that was not seen with FLAG-hGnRH-RI. Bright field images are shown in the second row from top. Scale bar = 10 µm.

Both the strong colocalization of hGnRH-RI and lamin A/C in HEK 293 and HTR-8/SVneo cells (Fig. 1F) and the identical pattern of colocalization observed to that of the nuclear mGluR5a and lamin A/C in HTR-8/SVneo cells (Fig. 2A) suggested that hGnRH-RI might be a nuclear GPCR associated with the nuclear membrane. This possibility led us to examine the protein sequence of hGnRH-RI for a putative nuclear localization signal (NLS). Using the Basic Local Alignment Search Tool (BLAST) to search for short nearly exact matches in the National Centre for Biotechnology Information protein database, we identified a putative monopartite NLS sequence, KKEKGKK (amino acid position 66–72) (Fig. 3A, Table 1). This sequence was conserved in other proteins characterized as nuclear localized proteins (Table 1). These include DNA topoisomerase II from *Candida* spp., pleiotrophic factor α2 (transcriptional regulator) from *Xenopus laevis*, HAI-2 related small protein (transcriptional regulator) from humans, and a number of other proteins from various species with putative nuclear functions such as the putative U3 small nuclear ribonucloprotein (snRNP) from *Leishmania major*. Next, using the DNASTAR Lasergene MegAlign program, we performed protein sequence alignments and observed that this sequence is fully conserved in the GnRH-RI protein expressed in the chimpanzee while in the mouse and rat, five of the seven residues in the primate sequence are found at the equivalent site (Table 1). This murine sequence (KRKKGKK), however, still contains a fully conserved monopartite NLS. A stretch of highly basic amino acid residues is also found in the chicken GnRH receptor (RKRRK) at the equivalent region in the first intracellular loop (Fig. 3A, Table 1), however, similar sequences are not located in any part of the mammalian type II GnRH-R (data not shown), a receptor that appears to be activated only by the type II GnRH.

**Table 1:** Proteins containing a putative monopartite NLS related to that found in the human gonadotropin releasing hormone receptor isoform 1.

Definition	Accession	Sequence	Amino Acid Position
Gonadotropin releasing hormone receptor isoform 1 [*Homo sapiens*]	NP_000397	KKEKGKK	66–72
U3 small nuclear ribonucloprotein (snRNP) [*Leishmania infantum* JPCM5]	XP_001466980	KKEKGKK	235–241
Type II DNA topoisomerase [*Candida tropicalis*]	BAB13749	KKEKGKK	288–294
Pleiotrophic factor-alpha1 [*Xenopus laevis*]	BAA07658	KKEKGKK	25–31
Alkylbase DNA N-glycosylase [*Cryptococcus neoformans* var. neoformans JEC21]	XP_570311	KKEKGKK	723–729
HAI-2 related small protein [*Homo sapiens*]	AAG43574	KKEKGKK	95–101
Gonadotropin releasing hormone receptor isoform 1 [*Pan troglodytes*]	XP_001163813	KKEKGKK	66–72
Gonadotropin releasing hormone receptor [*Mus musculus*]	NP_034453	KRKKGKK	66–72
Gonadotropin releasing hormone receptor [*Rattus norvegicus*]	NP_112300	KRKKGKK	66–72
Gonadotropin releasing hormone receptor [*Gallus gallus*]	NP_989984	RKRRK	69–73

### NLS deletion analysis: determining the effect on GnRH-RI spatial localization

To test whether the putative NLS located in the first intracellular loop of hGnRH-RI was required for the nuclear localization of the receptor, we performed single and multiple amino acid substitutions (mutating K and E to the non-charged G residue) and deletions as well as a full deletion of the KKEKGKK sequence in FLAG-hGnRH-RI and expressed the mutant receptor in HTR-8/SVneo cells. Surprisingly, we found that none of the mutations, including the full KKEKGKK deletion mutant, altered the spatial distribution of the receptor or frequency of cells expressing the receptor on the nuclear membrane (as assessed by lamin A/C colocalization) relative to the non-mutated receptor (Fig. 3B, data shown for the full putative NLS deletion mutant only). Interestingly, we found that although the full putative NLS deletion mutant still localized to the nuclear membrane, it showed a significantly diminished capacity to stimulate IP formation (in HEK 293 cells) following agonist treatment relative to its wild-type counterpart (Fig. 3C).

### K191 mutational analysis: determining the effect on GnRH-RI spatial localization

Next, based on previous data which revealed that the presence of a primate specific-residue, K191, in the hGnRH-RI contributed to an overall reduced PME, we investigated whether this residue also regulated nuclear membrane localization of hGnRH-RI. Our studies revealed that neither mutating the basic lysine residue to the acidic glutamic residue (E) nor deleting K191 led to any visual change on the cellular distribution of the receptor, particularly with respect to its nuclear membrane localization (Fig. 3D). This observation was further supported by IP formation data which revealed that there was no significant difference between the mutants, K191E and K191 deletion, compared to the wild-type FLAG-tagged human receptor. Nevertheless, both mutants showed a trend towards an increase in IP formation relative to the non-mutated receptor (Fig. 3E).

### Spatial characterization of the mouse GnRH-RI in HEK 293 and HTR-8/SVneo cells

Since the murine receptor lacks the K191 residue but contains a fully conserved monopartite NLS in the homologous position to the human NLS, we looked at the localization of the mouse FLAG-GnRH-RI in HEK 293 and HTR-8/SVneo cells. Our studies revealed that in both cell types, the mouse receptor was also expressed on the nuclear membrane (Fig. 3F, HEK 293 data not shown) like its hGnRH-RI counterpart (Fig. 1F). However, unlike the hGnRH-RI, the mGnRH-R was localized throughout the cytoplasm, and was weakly detected at the plasma membrane (Fig. 3F).

### Spatial characterization of the human-Xenopus GnRH-RI and Xenopus GnRH-RI in HEK 293 and HTR-8/SVneo cells: determining the effect of cytoplasmic tail sequences on GnRH-RI spatial localization

**Figure 4:**
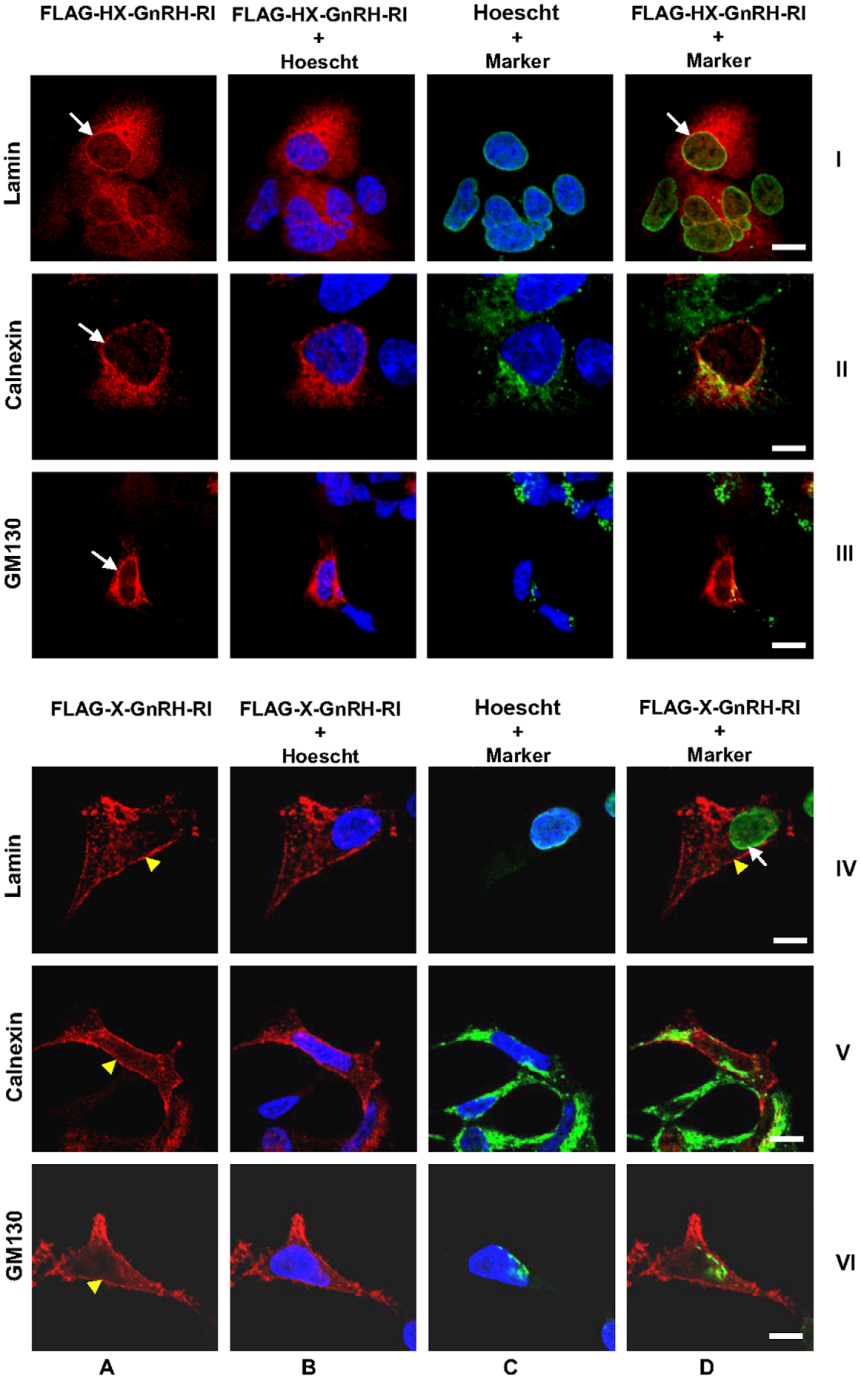
Spatial localization of FLAG-human-*Xenopus* (HX)-GnRH-RI and FLAG-*Xenopus* (X)-GnRH-RI in HEK 293 cells using organellar markers. Cells expressing FLAG-GnRH-RI (HX and X) were subjected to indirect immunofluorescent staining for the receptor (red) as well as the nuclear membrane, endoplasmic reticulum and Golgi (*all shown in green*). Colocalization is seen as yellow staining. Column A: receptor alone; Column B: receptor + Hoescht; Column C: organelle marker (lamin A/C, calnexin or GM130) + receptor; Column D: receptor + organelle marker. Column letters and roman numerals used as a coordinate system. HX-GnRH-RI immunoreactivity co-localized with the nuclear marker, lamin A/C, as seen by the yellow staining (I-D, *arrow*) as well as the endoplasmic reticulum marker, calnexin (II-D). Less colocalization was seen between the receptor and the Golgi, as shown by less yellow staining (III-D). X-GnRH-RI immunoreactivity strongly localized to the plasma membrane (yellow arrowheads) and weakly in cytoplasm. No visual co-localization detected with lamin A/C (IV-D, *arrow*), calnexin (V-D) or Golgi (VI-D). Scale bar = 10 µm.

To further explore the molecular determinants of GnRH-RI nuclear membrane localization, we assessed the contribution of cytoplasmic tail sequences to spatial localization. To conduct these studies we expressed the FLAG-tagged full-length *Xenopus* GnRH-RI and a FLAG-tagged chimeric human-*Xenopus* C-tail construct in both HEK-293 and HTR-8/SVneo cells and determined their spatial localization. Our findings revealed that in both cell types in the absence and presence of agonist, the chimeric GnRH-RI sequences displayed a nuclear localization pattern identical to the full-length wild-type human GnRH-RI sequences and this was found in greater than 95% of all cells (Table 2, Fig. 4) expressing the chimeric construct. Interestingly, however, when the full-length wild-type *Xenopus* sequences were expressed, the receptor was mainly found at the plasma membrane and evenly distributed intracellularly with less than 5% of the cells showing any nuclear localization (Table 2, Fig. 4). This was consistent for both cell types in the presence and absence of agonist.

**Table 2:** Frequency of subcellular distribution of human-*Xenopus* chimeric GnRH-R (HX-GnRH-R) and full-length *Xenopus* GnRH-R (X-GnRH-R) in HEK 293 cells as assessed by confocal microscopy.

Receptor	Plasma Membrane (%)^1^	Nuclear Membrane (%)^1^	ER (%)^1^
HX-GnRH-R	0	97	100
X-GnRH-R	100	4	0

1 Data based on the examination of 100 cells expressing each receptor subtype.

### The nuclear-localized GnRH-RI is functional

**Figure 5:**
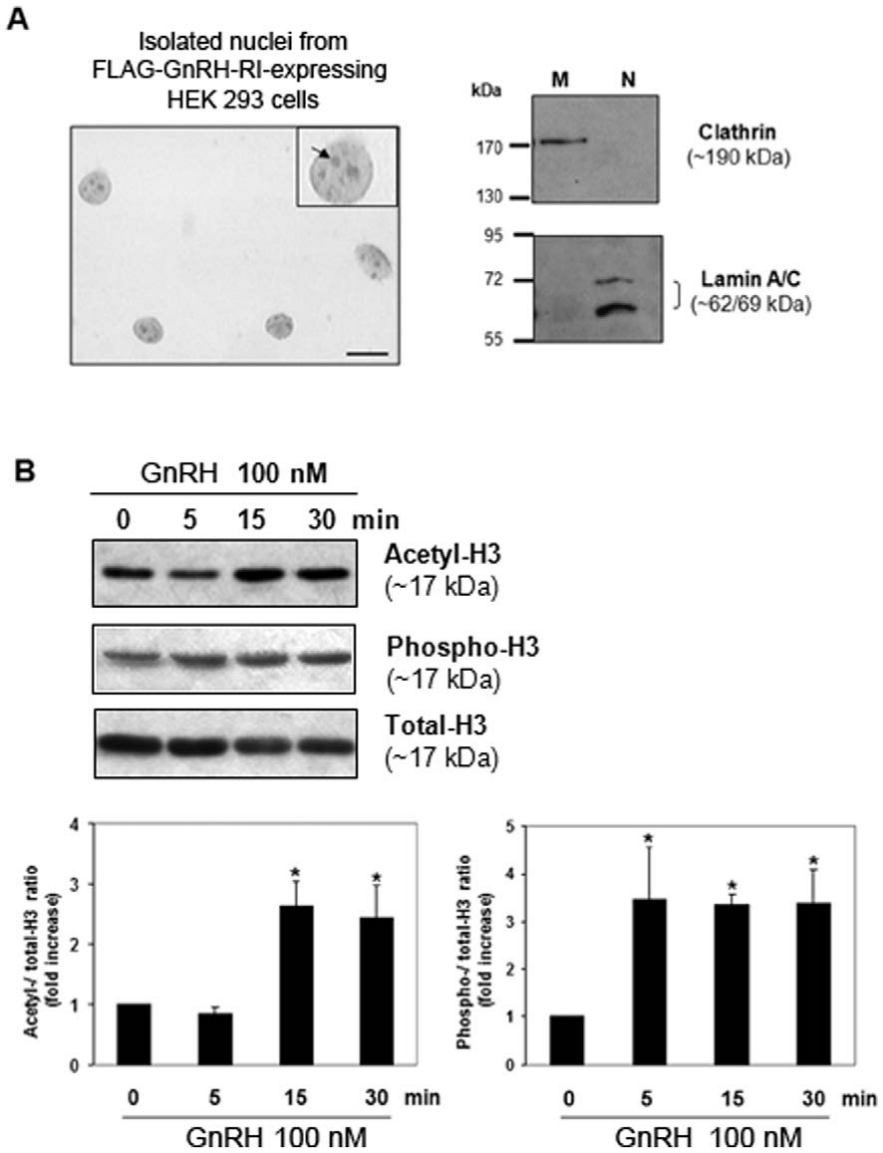
Effect of GnRH stimulation on Histone 3 acetylation/phosphorylation levels in isolated nuclei from HEK 293 transiently transfected with FLAG-GnRH-RI. (A) Purity assessment of nuclei preparations used in functional assays by bright field microscopy and Western blotting. Left panel: Photomicrograph of a typical nuclei preparation following trypan blue staining. Scale bar = 10 µm. Insert: digital magnification of a single nucleus showing the presence of nucleoles (arrow). Right panel: Immunodetection of the membrane and nuclear markers clathrin and lamin A/C respectively in membrane and isolated nuclei fractions from FLAG-GnRH-R-transfected HEK 293 cells. (B) Time course of Histone H3-Lys9/Lys14 acetylation and -Ser10 phosphorylation in GnRH-stimulated nuclei by Western blot analysis. A representative Western blot is shown. Densitometric values of acetylated and phosphorylated Histone H3 levels were normalized to those of total Histone H3 and the relative intensity ratios were expressed in-fold increase over time zero. Data are means ± S.E.M of four independent experiments. *P<0.05 vs control time zero. Statistical analyses performed using one-way ANOVA followed by Dunnet's ad hoc test.

Once we were confident that the human and mouse GnRH-RI were expressed at the nuclear membrane we determined whether the nuclear membrane localized hGnRH-RI was functional. Since these studies required a large number of nuclei we used HEK 293 cells since they were more efficiently transfected than the HTR-8/SVneo cells. HEK 293 cells were transfected with hGnRH-RI and nuclei were isolated under detergent-free conditions. Nuclei integrity and purity were determined visually and by immunoblotting for subcellular markers. These analyses confirmed that nuclei preparations of very high integrity and purity were obtained using our isolation method (Fig. 5A). Based on recent studies that show the nuclear bradykinin B2 receptor is functionally coupled to histone acetylation [20] we assayed for GnRH stimulated histone H3 acetylation and found that GnRH triggered a significant increase in acetylation following 15 and 30 minutes of stimulation (Fig. 5B). We then assayed for H3 phosphorylation and also found that GnRH triggered a significant increase in phosphorylation following 5, 15 and 30 minutes of stimulation (Fig. 5B).

## Discussion

It has been almost three decades since Millar's laboratory demonstrated the presence of the GnRH receptor on rat anterior pituitary nuclei through the use of radiolabeled GnRH agonists [21] and about a decade since Conn's laboratory proposed the idea that the GnRH receptor has evolved towards reduced plasma membrane expression [8]. Additionally, there have been at least four other studies that provide evidence that GnRH-R is detectable at the nucleus. Following Millar's findings in 1983, Morel et al. [22] in 1987 demonstrated that extensive radiolabeled GnRH accumulated over the nucleus and nuclear membrane of radioligand treated rat gonadotrophs. The assumption was that the label was bound to the rat GnRH-R at these sites. Subsequently, in an immunohistochemical analysis of ligand treated hamster pancreatic cancer cells, Szende *et al.* [23] demonstrated that GnRH-R accumulated in the nucleus. In cell fractionation studies using human breast cancer tissue Mangia et al. [24] demonstrated that GnRH-R was associated with the nuclear compartment while more recently, Meethal et al. [25] demonstrated through immunohistochemical studies that in the nematode *C. elegans*, GnRH-R was located in the nucleus. These studies provided evidence that endogenously expressed GnRH-R from the rat, hamster, nematode and human was detectable at the nucleus. While these were informative studies, the image quality could not allow for conclusive proof that the receptor is nuclear. Furthermore, they provided no evidence that the nuclear-localized GnRH-R is functional. Nevertheless, they do provide support for our findings which clearly reveal that the human and mouse GnRH-RI are expressed strongly at the nuclear membrane.

Our spatial observations are consistent with those previously reported by Sedgley et al. [26] who also observed that in mammary (MCF7) cells the wild-type human GnRH-RI is inefficiently expressed at the plasma membrane. Our study is also consistent with previous biochemical data [8], [11], [27]–[31] that suggest both the wild-type and naturally occurring mutant human GnRH-RI are expressed inefficiently at the plasma membrane and are retained intracellularly, both in the presence and absence of agonist. The mouse homologue is also expressed at similar levels in both cell lines and our spatial data again support previous biochemical data that reveal, unlike the mouse receptor, a large fraction of the human receptor is retained in the ER [9], [10], [27]. Both the human and mouse receptors additionally show marked expression at the nuclear membrane as revealed by colocalization with lamin A/C. Lamin A/C, a type V nuclear lamin, is an Intermediate Filament protein that is a component of the nuclear lamina, a fibrous layer on the nucleoplasmic side of the inner nuclear membrane which is suggested to provide a framework for the nuclear envelope and may interact with chromatin. Additionally, both receptors are also expressed at other intracellular sites; weakly in the Golgi and strongly in the ER. Brothers et al. [32] recently demonstrated through immunoprecipitation assays that human GnRH-RI physically interacts with calnexin in COS-7 cells, where it is suggested to retain misfolded receptor molecules while routing correctly folded molecules to the plasma membrane.

The observation that hGnRH-RI is inefficiently expressed at the plasma membrane and strongly expressed intracellularly is proposed to be the result of a progressive and convergent evolutionary trend of the GnRH-R [11]. Potentially, intracellular retention of the receptor creates and provides a source of GnRH-RI needed for rapid availability without transcription or translation. A similar mechanism might also regulate the human δ opioid receptor since a study by Petäjä-Repo et al. [33] demonstrated that permeable agonists and antagonists of the receptor allowed post-translational processing and increased export of the ligand-stabilized receptor from the ER to the cell surface. Other reports indicate that receptors such as GluR1, α_1_D adrenoreceptor, odorant and luteinizing hormone receptors are also inefficiently expressed at the plasma membrane [34]–[38], thus suggesting that reduced receptor trafficking may represent a more widespread mechanism for regulating protein availability [11]. In this study we suggest that the mammalian GnRH-R is evolving towards greater intracrine signaling in cells that express both the receptor and ligand, such as the HTR-8/SVneo. Intracrine signaling would appear to offer the cell more efficient and rapid control of a signaling pathway. This is in contrast to autocrine signaling which emanates at the plasma membrane, incorporates additional signaling steps and may require more energy only to eventually culminate in the same final cellular response as a pathway that was initiated intracellularly.

To date, there is an increasing number of GPCRs identified as nuclear GPCRs [39] and perhaps the best characterized nuclear localized GPCR is the angiotensin (AT_1_) receptor. The nuclear localization of the AT_1_ receptor is induced by angiotensin II [40], [41] and studies have uncovered angiotensin II-binding sites in the nucleus and have also demonstrated the angiotensin II-induced transcription of renin and angiotensinogen mRNA [42]. Other GPCRs that are localized to the nucleus include the prostaglandin EP_1_ [43], EP_3_, and EP_4_ receptors [44], parathyroid hormone receptor [45], [46], metabotropic glutamate mGluR1a and 5a receptors [16], [17], endothelin ETA and ETB receptors [47], apelin and bradykinin (B_2_) receptors [48]. Some of these receptors are located in the nucleoplasm and/or at the nuclear membrane [17].

The AT_1_ receptor was reported to traffic to cell nuclei by the presence of an NLS sequence in its 8^th^ helix (membrane proximal C-terminal sequences) [41]. Subsequently NLS sequences have been identified in the majority of GPCRs observed to have nuclear localization. NLS sequences identified in GPCRs are generally found in the 8^th^ helix, however, in the apelin receptor it is located in the third intracellular loop [48]. The human (KKEKGKK) and mouse (KRKKGKK) GnRH-RI putative NLS sequences are located in the first intracellular loop and form a stretch of basic amino acid residues that contains the monopartite consensus sequence K-K/R-X-K/R [49] which strongly resembles both well established and putative NLS sequences identified in nuclear localized proteins like DNA topoisomerase II from Candida spp., pleiotrophic factor α2 (transcriptional regulator) from *Xenopus laevis*, HAI-2 related small protein (transcriptional regulator) from humans, and a number of other proteins from various species with putative nuclear functions such as the putative U3 small nuclear ribonucloprotein (snRNP) from Leishmania major.

Based on the presence of this sequence in proteins with established nuclear function, we hypothesized that the human and mouse GnRH-RI contained a functional NLS. However, mutagenesis studies revealed that in both HEK 293 and HTR-8/SVneo cells, the hGnRH-RI mutant lacking the KKEKGKK sequence was still strongly expressed at the nuclear membrane. This observation leads us to consider that this is not the functional NLS or that it might not be the only NLS, thus its deletion may not be sufficient to disrupt nuclear membrane localization. Also, it is possible that while this is the NLS, additional flanking residues must be deleted to disrupt localization. Finally, we must consider that nuclear membrane localization is NLS-independent. Since the hGnRH-RI is found on the nuclear membrane, as opposed to within the nucleoplasm an NLS may not be required. This idea is supported by evidence suggesting that the endothelin receptor subtypes A and B, which have a perinuclear distribution, localize to the nuclear membrane via *de novo* synthesis and retrograde transport [47]. Further investigations are required to test whether this mechanism accounts for hGnRH-RI localization at the nuclear membrane.

To further explore the regulation of hGnRH-RI nuclear localization, we examined the spatial localization of the human-*Xenopus* C-tail chimeric GnRH-RI and full-length *Xenopus* GnRH-RI in HEK 293 and HTR-8/SVneo cells. Our results revealed that the full-length *Xenopus* receptor, in the absence of any human sequences, was hardly detected at the nuclear membrane but strongly expressed on the plasma membrane. The data suggest that nuclear localization is strongly determined by sequences in the human receptor. It is possible that these sequences are poorly conserved in the *Xenopus* receptor, thus, the receptor is infrequently expressed at the nuclear membrane. It also seems that in HEK 293 cells, *Xenopus* cytoplasmic tail sequences are not sufficient to *visibly* increase plasma membrane expression of the hGnRH-RI. This observation was unexpected since studies have demonstrated that cytoplasmic tail sequences potentiate the plasma membrane expression of GPCRs [50], [51], including that of the human GnRH-RI [26]. The differences observed in our HEK 293 cell-based study compared to the MCF7 cell-based study by Sedgley et al. [26] suggests that nuclear localization might be cell type-dependent. This conclusion is in full agreement with those of Finch et al. [52] who show variations in surface expression of the human GnRH-RI and chimeric HX-GnRH-RI among various cell lines reported to endogenously express GnRH-RI.

While it is not definite proof, failure to observe a strong and frequent nuclear localization of the overexpressed *Xenopus* FLAG-GnRH-R strengthens the conclusion that FLAG-hGnRH-RI is not localized to the nuclear membrane due to its overexpression. This idea is further supported by the earlier observation that overexpression of the well-established nuclear GPCR, (FLAG-tagged) mGluR5a, is localized to the nuclear membrane while overexpression of the (FLAG-tagged) β2AR, a GPCR determined not to be nuclear localized, is only detected at the plasma membrane.

While our results clearly indicate that GnRH-RI is expressed at the nuclear membrane, our study could not report on the precise orientation of the receptor at the nuclear membrane. In the case of the nuclear membrane localized EP_1_ receptor, the authors show by electron microscopy that the receptor is located at both the inner and outer nuclear membranes [43]. In both locations, the receptor would have easy access to its ligand since the prostanoid synthesis enzymes are located at both the inner and outer nuclear membranes [53]. In the case of nuclear membrane localized mGluR1a and 5a, it is reported that the receptors are oriented with their N-terminal, ligand-binding domain within the lumen of the nuclear envelope; hence, agonists must cross both the plasma and nuclear membranes to access binding domains [17], [54]. In addition to other mechanisms that may exist, ligand activation of nuclear mGluRs expressed in HEK 293 cells is achieved via endogenous sodium-dependent transporters and cystine glutamate exchangers that mediate the rapid delivery of quisqualate or glutamate into the cell and into the nuclear lumen [17], [54]. With respect to the activation of GnRH-RI, extracellular GnRH might also be routed to the nuclear membrane employing a similar mechanism as described for the mGluRs. In addition, since cytotrophoblasts, such as HTR-8/SVneo, express both GnRH-RI and its ligands [1], [55], it remains a possibility that ligands produced intracellularly can somehow be routed to the nuclear membrane to cause an activation of GnRH-RI and thus trigger off an intracrine signaling pathway.

Our clear demonstration that GnRH-RI is expressed strongly at the nuclear membrane is a powerful finding as it raises questions about its function at this location. Recently, members of our collaborative group demonstrated that the nuclear bradykinin B2 receptor positively regulates H3 acetylation [20] leading us to explore the possibility that GnRH-RI also regulates histone H3 acetylation. Our results showed that it does. Additionally, we also showed that nuclear GnRH-RI also potentiates histone phosphorylation; the first demonstration that a nuclear GPCR can regulate H3 phosphorylation. Overall, our results indicate that GnRH-RI might play a more direct role in regulating gene expression than previously appreciated and while it was not within the scope of the present study our ongoing studies are aimed at determining which genes are regulated by nuclear GnRH-RI. Our present finding that the human GnRH-RI receptor is a functional nuclear GPCR opens up many exciting possibilities on novel cellular functions associated with this unique and clinically important GPCR and confirms the significance of findings first made three decades ago [21].

## Materials and Methods

### Materials

Restriction enzymes were obtained from Promega (Madison, WI, USA) and New England Biolabs Inc. (Pickering, ON, Canada). HEK 293 cells were from American Type Culture Collection (ATCC, Manassas, VA, USA). HTR-8/SVneo cells were a gift from Dr. Peeyush Lala from the University of Western Ontario. Fetal bovine serum (FBS), collagen, rabbit anti-FLAG antibody and Buserelin were purchased from Sigma Aldrich Inc. (Oakville, ON, Canada), mouse anti-lamin A/C, and mouse anti-calnexin from AbCam (Cambridge, MA, USA), mouse anti-GM130 from Transduction Laboratories (BD Biosciences, Mississauga, ON, Canada), murine anti-clathrin monoclonal antibody from Santa-Cruz Biotechnology (Santa-Cruz, CA, USA), rabbit anti-Histone H3 and rabbit anti-phospho-Histone H3 (Ser10) polyclonal antibodies from Cell Signaling (Danvers, MA, USA) and rabbit anti-acetyl-Histone H3 polyclonal (Lys9 and Lys14) antibody from Upstate Biotechnology (Lake Placid, NY, USA). Secondary antibodies conjugated to Alexa Fluors, Hoechst dye, pcDNA3.1/Hygro(+) vector, media and media supplements were acquired from Invitrogen (Burlington, ON, Canada). Quisqualate was from Tocris Biosciences (Avonmouth, Bristol, UK). All other biochemical reagents and culture products were purchased from BioShop, Fisher Scientific and VWR.

### WT and mutant GnRH-RI expression constructs

A 987-bp cDNA fragment containing the complete coding sequence of human GnRH-RI (accession number NM_000406.2) was obtained by RT-PCR from the QUICK-Clone™ cDNA library (Clontech Laboratories Inc., CA, USA). The FLAG sequence was introduced at the amino terminus of hGnRH-RI by PCR. FLAG-hGnRH-RI was then cloned into the pcDNA3.1/Hygro(+) vector using *Nhe*1 and *Not*1 sites. The untagged receptor was created by inserting an *Eco*RI site with a Kozak sequence between the FLAG tag and hGnRH-RI by PCR and then subcloning hGnRH-RI into the pcDNA 3.1 Neo vector using *Eco*RI and *Not*1. Human GnRH-RI-GFP was made as previously reported [30], [56].

To create the NLS deletion mutant, K191deletion mutant, K191E mutant, FLAG-hGnRH-RI was subjected to site-directed mutagenesis using the QuickChange kit following manufacturer's instructions (Stratagene, CA, USA). The putative NLS deletion mutant was created by performing two sequential deletions; first removing K66 and K67, followed by the deletion of E68, K69, G70, K71 and K72. The following oligonucleotides were used:

Putative NLS deletion mutants:

K66del/K67del FWD CTTCAGAAGTGGACACAGΔGAGAAAGGGAAAAAGCTC

K66del/K67del REV GAGCTTTTTCCCTTTCTCΔCTGTGTCCACTTCTGAAG

E68del/K69del/G70del/K71del/K72del FWD GTTGAAACTTCAGAAGTGGACACAGΔCTCTCAAGAATGAAGC

E68del/K69del/G70del/K71del/K72del REV GCTTCATTCTTGAGAGΔCTGTGTCCACTTCTGAAGTTTCAAC

The lysine 191 mutants were created in a single mutagenesis reaction using the following oligonucleotides:

Lysine 191 mutants:

K191E FWD GCAGACAGCTCTGGACAGACAGAAGTTTTCTCTCAATGTG

K191E REV CACATTGAGAGAAAACTTCTGTCTGTCCAGAGCTGTCTGC

K191deletion FWD GCTCTGGACAGACAΔGTTTTCTCTCAATGTGTAACACAC

K191deletion REV GTGTGTTACACATTGAGAGAAAACΔTGTCTGTCCAGAGC

A 984-bp cDNA fragment containing the complete coding sequence of mouse GnRH-RI (accession number NM_010323.1) was isolated by RT-PCR using RNA from the pituitary of a female 129/Sv mouse (Charles River). *Bam* HI and *Not* I sites were cloned onto the 3′ and 5′ end of the cDNA construct, respectively. To create the FLAG-tagged mouse GnRH-RI construct, the human GnRH-RI was removed from the FLAG-pcDNA 3.1 Hygro+ vector using *Bam*HI and *Not*I and the mouse GnRH-RI ORF was ligated in.

An 1107 bp fragment corresponding to the type I *Xenopus laevis* GnRH-R open reading frame (accession number AF172330) and an 1149 bp chimeric fragment corresponding to the first 326 amino acids of the type I human GnRH-R (978 bps) fused to 56 amino acids corresponding to the type I *Xenopus* GnRH-R tail sequences (171 bps) were subcloned from the pCR3 vector (Invitrogen)[57], FLAG-epitope tagged on their N-terminus and cloned as a *Nhe*I-*Not*I fragment into a home-made cloning vector based on the pEGFP (Clontech) vector backbone.

The sequence integrity of all constructs described in this study was verified for sequence integrity by nucleotide sequencing.

### Cell culture and Immunofluorescence

HEK 293 cells were cultured in MEM supplemented with 10% (v/v) FBS, 1% (v/v) non-essential amino acids and gentamicin (5 µg/ml). HTR-8/SVneo cells were maintained in RPMI supplemented with 10% FBS, 1% non-essential amino acids, 1% glutamax, 1% sodium pyruvate and 1% penicillin/streptomycin (v/v). Both cell lines were maintained at 37°C in a humidified atmosphere containing 5% CO_2_. HEK 293 cells were transiently transfected with 5 µg of DNA using a modified calcium phosphate method as previously described [58] or using the ExGen 500 transfection reagent (MBI Fermentas, Canada). After transfection (18 h), the cells were incubated with fresh medium, allowed to recover for 6 hours and reseeded onto 18 mm collagen-coated glass coverslips. Cells were allowed to grow an additional 18 h prior to experimentation. HTR-8/SVneo cells were transiently transfected with 10 µg of DNA by electroporation using the Bio-Rad Gene Pulser Xcell System with 0.2 mm electroporation cuvettes (BioRad) and the CHO preset protocol. Post transfection, cells were allowed to recover for 24 hours before being plated on 18 mm collagen coated glass coverslips in 12 well plates. 42–44 hours post-transfection, both HTR-8/SVneo and HEK 293 cells were washed twice with Hanks' balanced salt solution (HBSS: 1.2 mM KH2PO_4_, 5 mM NaHCO_3_, 20 mM HEPES, 11 mM glucose, 116 mM NaCl, 4.7 mM KCl, 1.2 mM MgSO_4_, 2.5 mM CaCl_2_, pH 7.4) and either stimulated [100 nM Buserelin for GnRH-RI and 100 µM Quisqualate for mGluR5a] for two hours or left untreated. Cells were fixed and permeabilized using 4% formaldehyde and 0.2% Triton-X in HBSS for 20 minutes, washed 4 times with HBSS and blocked with HBSS-3% BSA for 30 minutes before the addition of primary antibody. Cells were incubated with primary antibody overnight at 4°C at the following concentrations: polyclonal anti-FLAG 1∶2000, monoclonal anti-calnexin 1∶100 (to detect endoplasmic reticulum), monoclonal anti-GM130 1∶100 (to detect the Golgi apparatus), monoclonal anti-lamin A/C 1∶10 (to detect the inner nuclear membrane). Coverslips were then washed 4 times with HBSS and blocked for an additional 30 minutes in HBSS containing 3% BSA before incubation with secondary antibody for 45 minutes at room temperature. Goat anti-mouse antibody conjugated to AlexaFluor 488 was used at a dilution of 1∶250, while goat anti-rabbit antibody conjugated to AlexaFluor 568 was used at 1∶1200. Cells were washed 4 times with HBSS and then counter-stained using Hoechst at 1∶50000 (v/v) for 7 minutes to detect DNA. After 4 additional washes with HBSS, cells were mounted onto glass slides and allowed to dry overnight at room temperature. Confocal analysis was performed on an Olympus Fluoview 1000 laser scanning confocal microscope using either the 60X Plan Apochromat 1.42 oil objective or the 100x Plan superapochromat 1.4 Oil objective. Colocalization studies were performed using multiple excitation (405, 488, 559) and emission (band pass 425–475, 500–545 nm and 575–675 nm for Hoechst, AlexaFluor 488 and AlexaFluor 568 respectively) filter sets. Multi-colour images were acquired in the sequential acquisition mode to avoid cross-excitation.

### Nuclei isolation for immunofluorescence studies

Nuclei were isolated using the Sigma Nuclei EZ Prep Nuclei Isolation Kit using a modified protocol. In brief, nuclei were isolated from HEK 293 cells expressing FLAG-GnRH-RI, FLAG-mGluR5a or FLAG-β2AR approximately 42–44 hours post-transfection. Cells were stimulated with agonist for 2 hours or left untreated, washed twice with ice cold PBS and then lysed using 15 strokes of a 15 ml Dounce homogenizer using the tight pestle. The lysate was mixed with a 1.8 M sucrose solution and then layered over 10 ml of 1.8 M sucrose solution in a 40 ml Beckman centrifuge tube (Beckman Coulter Inc., Fullerton, CA, USA). Tubes were spun at 12900 rpm in a swinging bucket rotor for 85 minutes to pellet the nuclei. The supernatant was removed and nuclei were washed and resuspended in storage buffer (provided). A sample of nuclei was plated on 18 mm collagen coated coverslips in 12 well plates and fixed and immunostained as described above.

### Nuclei isolation for functional studies

Nuclei isolation technique was adapted from [59]. HEK 293 cells were washed with ice-cold PBS (10 ml/20 cm plate), gently scraped on ice in 5 ml PBS/1 mM EDTA and centrifuged at 500 g for 5 min at 4°C. Pelleted cells were resuspended in 1 ml hypotonic lysis buffer (10 mM Tris-HCl pH 7.4, 5 mM KCl, 3 mM MgCl_2_ and protease inhibitor cocktail (Roche), allowed to swell on ice for 1 h then homogenized (100 gentle strokes) using a Dounce tissue grinder (tight pestle; Bellco Glass). Nuclear samples were diluted to 15 ml with lysis buffer, centrifuged (800 g, 8 min at 4°C) and resuspended in 5 ml stimulation buffer (20 mM Hepes pH 7.4, MgCl_2_ 3 mM, KCl 20 mM, CaCl_2_ 200 nM, ATP 25 µM and glycerol 10%) for visual inspection and counting of nuclei. Protein concentration was determined using the BCA method. Nuclei were stored at −80°C unless otherwise stated. Purity of subcellular fractions was further substantiated by means of immunological methods using the specific organelle marker antigens clathrin (plasma membrane) (1∶250 dilution) and lamin A/C (nuclei) (1∶100 dilution).

### Acid extraction of histone and Western blotting of Histone H3

HEK 293 nuclei (6–10×10^6^ nuclei/assay) were resuspended in stimulation buffer (20 mM Hepes pH 7.4, MgCl_2_ 3 mM, KCl 20 mM, CaCl_2_ 200 nM, ATP 25 µM and glycerol 10%), allowed to pre-equilibrate at 37°C for 15 min with gentle shaking, then challenged with 100 nM of GnRH for 0, 5, 15 and 30 min. Reaction was terminated by freezing samples in liquid nitrogen. Acid extraction of histones was performed according to [20]. Briefly, nuclei were resuspended in lysis buffer (Tris–HCl 50 mM pH 8, MgCl_2_ 5 mM, KCl 25 mM containing HCl 0.2 N, 1 mM NaF, 1 mM Na_3_VO_4_, 2.5 mM sodium butyrate and Roche's EDTA-free protease inhibitor cocktail), and incubated on ice for 30 min. After centrifugation at 11,000 g for 15 min, 1 ml of cold acetone was added to the supernatant and placed at −20°C for 4 h. Precipitated proteins were collected by centrifugation at 11,000 g for 15 min. Pellets were washed twice with cold acetone, dried and resuspended in 25 µl of water containing 1 mM NaF, 1 mM Na_3_VO_4_, 2.5 mM sodium butyrate and protease inhibitors cocktail. Acid proteins including histones were quantified using the BCA method and 5 µg were loaded on a 15% polyacrylamide gel and analyzed by Western blotting using an anti-acetylated (1∶3,000), anti-phosphorylated (1∶5,000) or anti total histone H3 (1∶2,500) antibody.

### Western Blotting

Proteins were analyzed by Western blotting and immunoblots were visualized by chemiluminescence using an ECL kit (GE). Densitometric analysis of autoradiograms was performed using ImagePro 5.1 software.

### Inositol Phosphate Formation

Inositol lipids were radiolabeled by incubating cells overnight with 1 µCi/ml [^3^H]myo-inositol in Dulbecco's modified Eagle's medium. Unincorporated [^3^H]myo-inositol was removed by washing the cells with HBSS. Cells were preincubated for 1 h in HBSS at 37°C and then preincubated in 500 µl of the same buffer containing 10 mM LiCl for an additional 10 minutes at 37°C. Next, the cells were incubated in either the absence or the presence of 100 nM Buserelin for 2 hours at 37°C. The reaction was stopped on ice by adding 500 µl of 0.8 M perchloric acid and then neutralized with 400 µl of 0.72 M KOH, 0.6 M KHCO_3_. The total [^3^H]inositol incorporated into the cells was determined by counting the radioactivity present in 50 µl of the cell lysate. Total inositol phosphate was purified from the cell extracts by anion exchange chromatography using Dowex 1-X8 (formate form) 200–400 mesh anion exchange resin. [^3^H]Inositol phosphate formation was determined by liquid scintillation using a Wallac LKB 1211 RackBeta liquid scintillation counter. The means ± S.E. are shown for the number of independent experiments indicated in the figure legends. GraphPad Prism software was used to analyze data for statistical significance. Statistical significance was determined by Student's t-test or one-way analysis of variance followed by Dunnet's ad hoc test. P-values <0.05 were considered to be significant.
